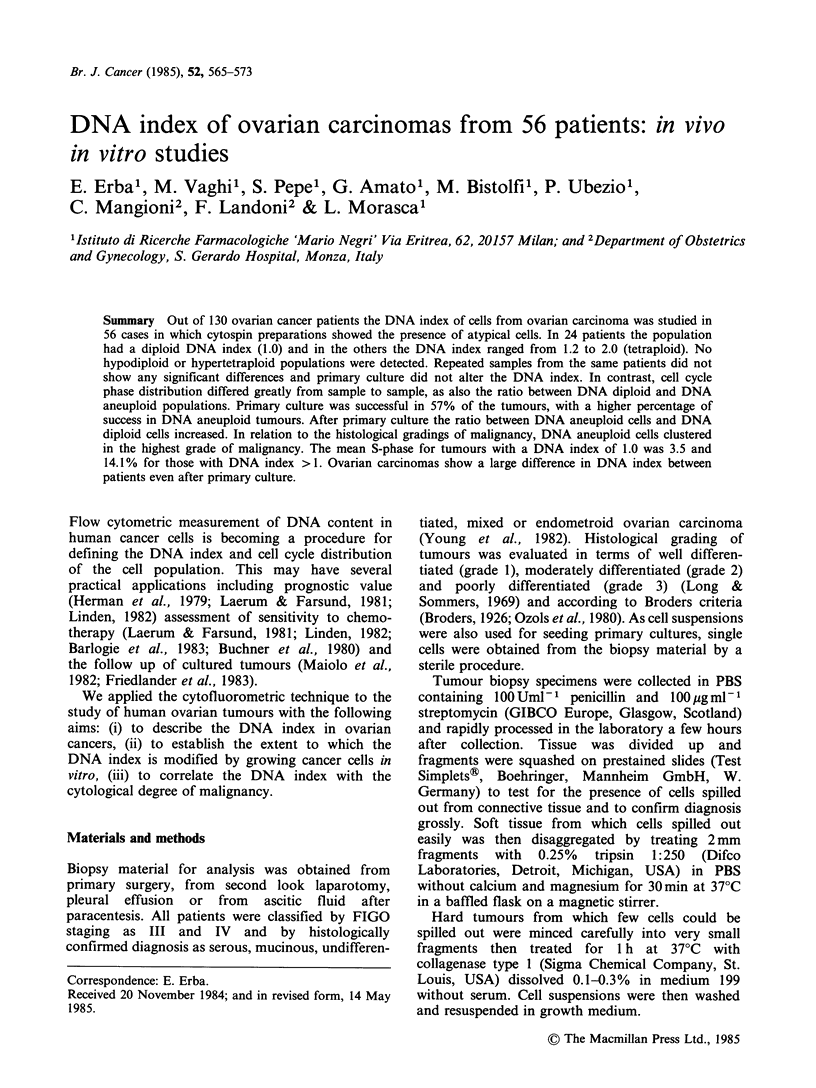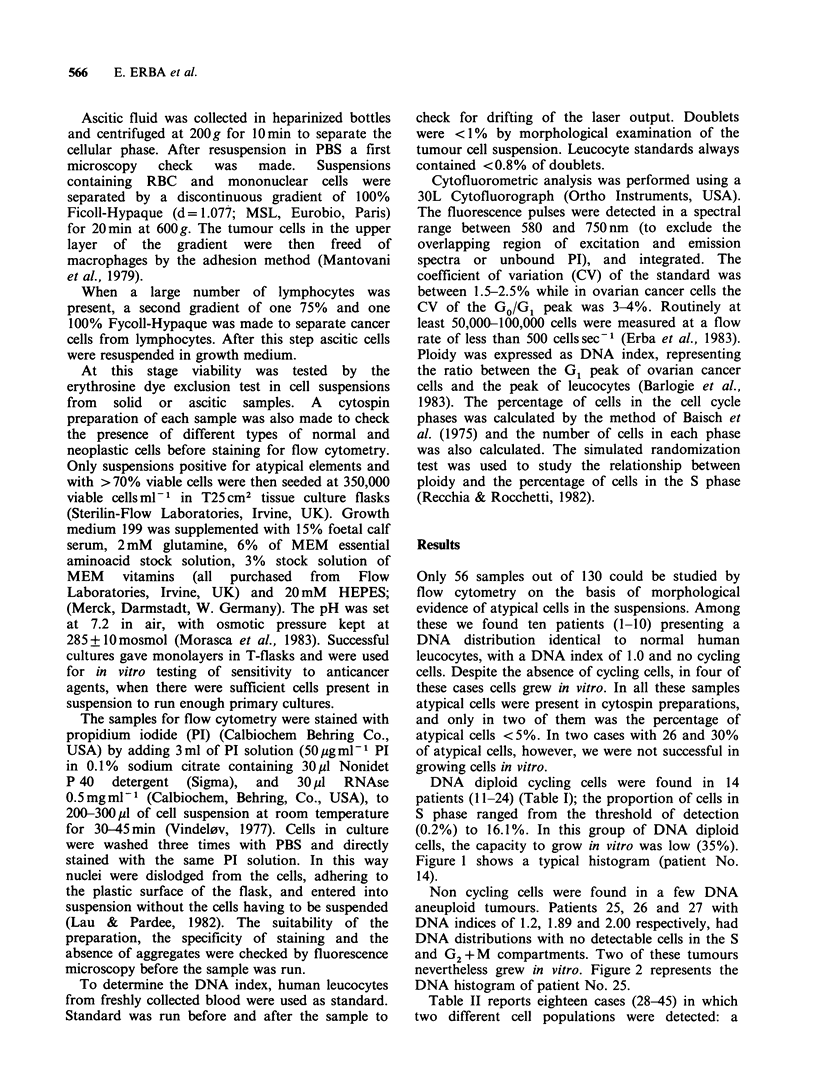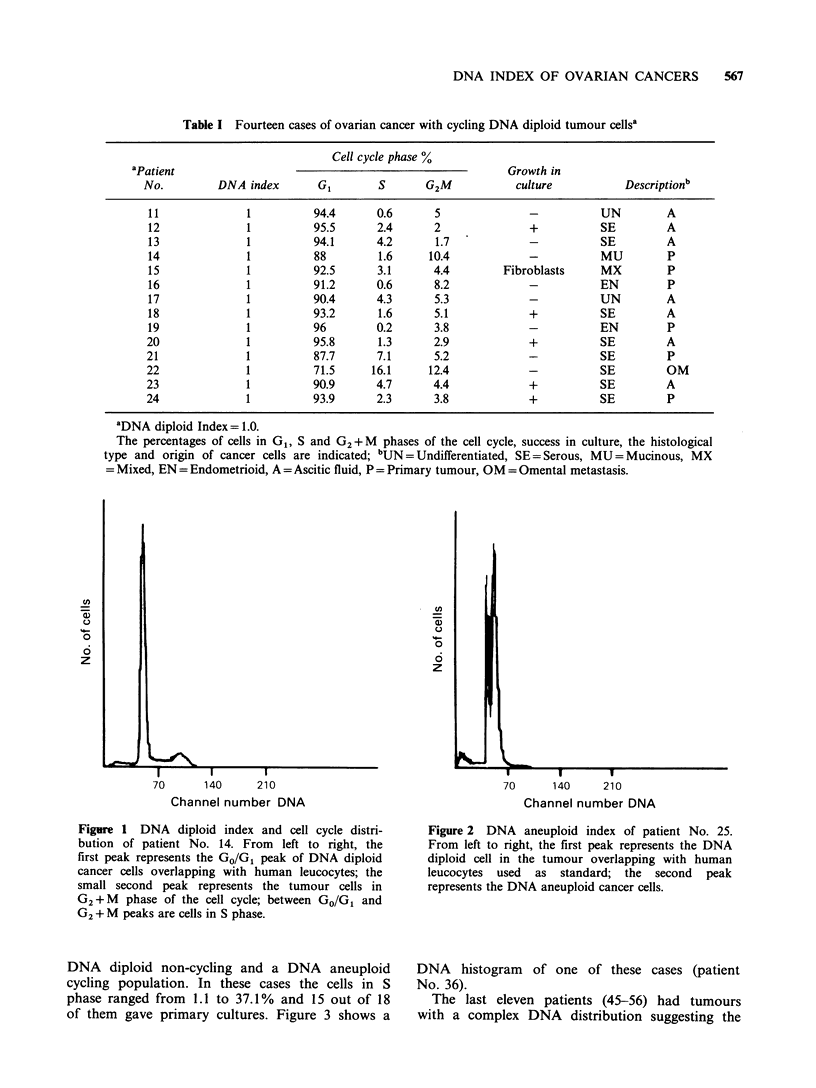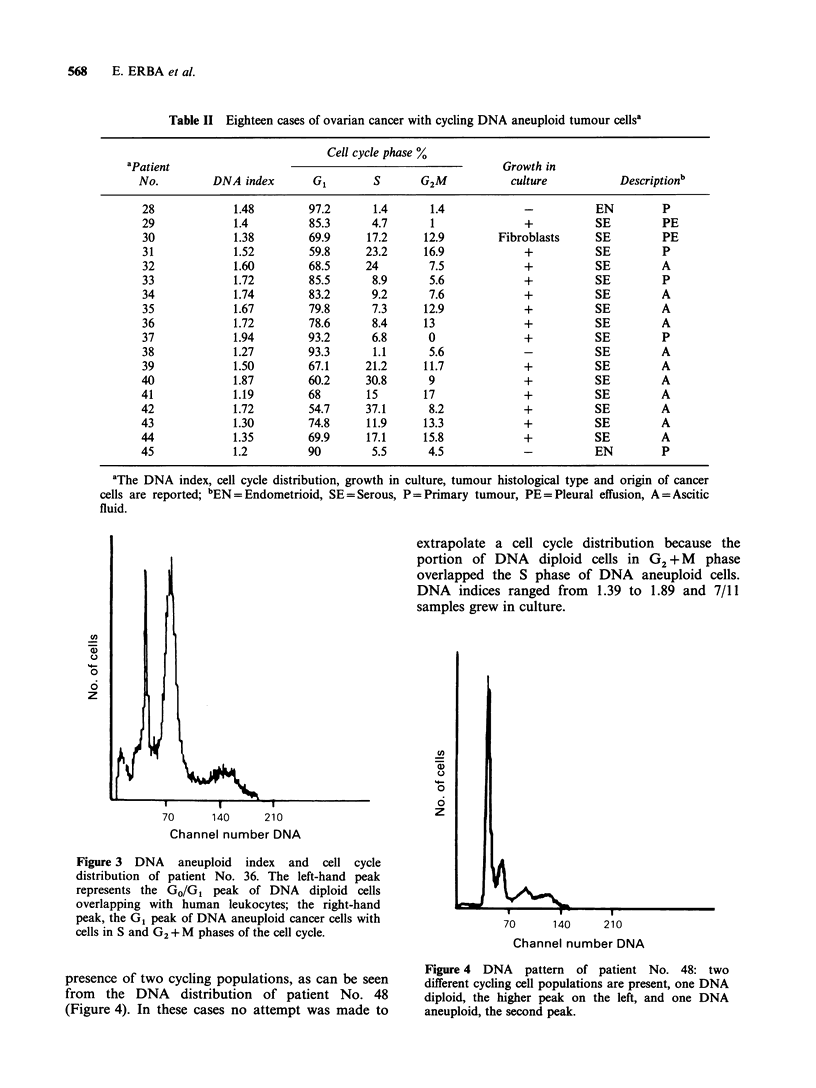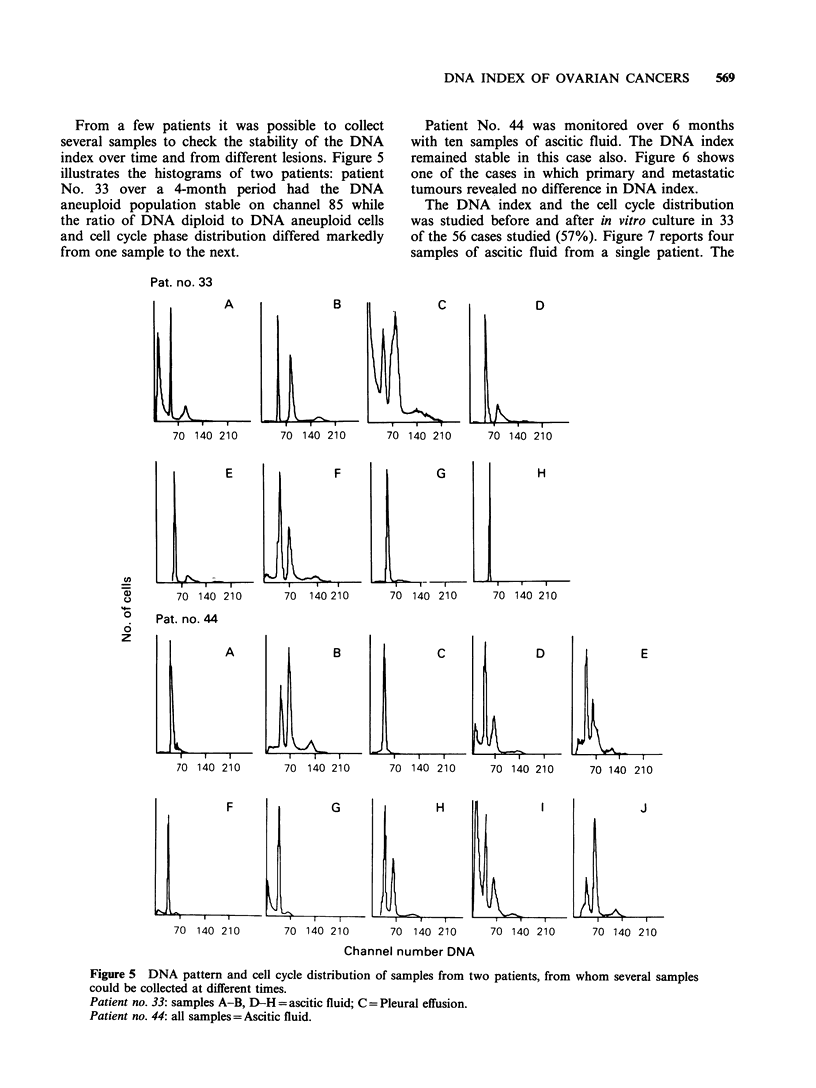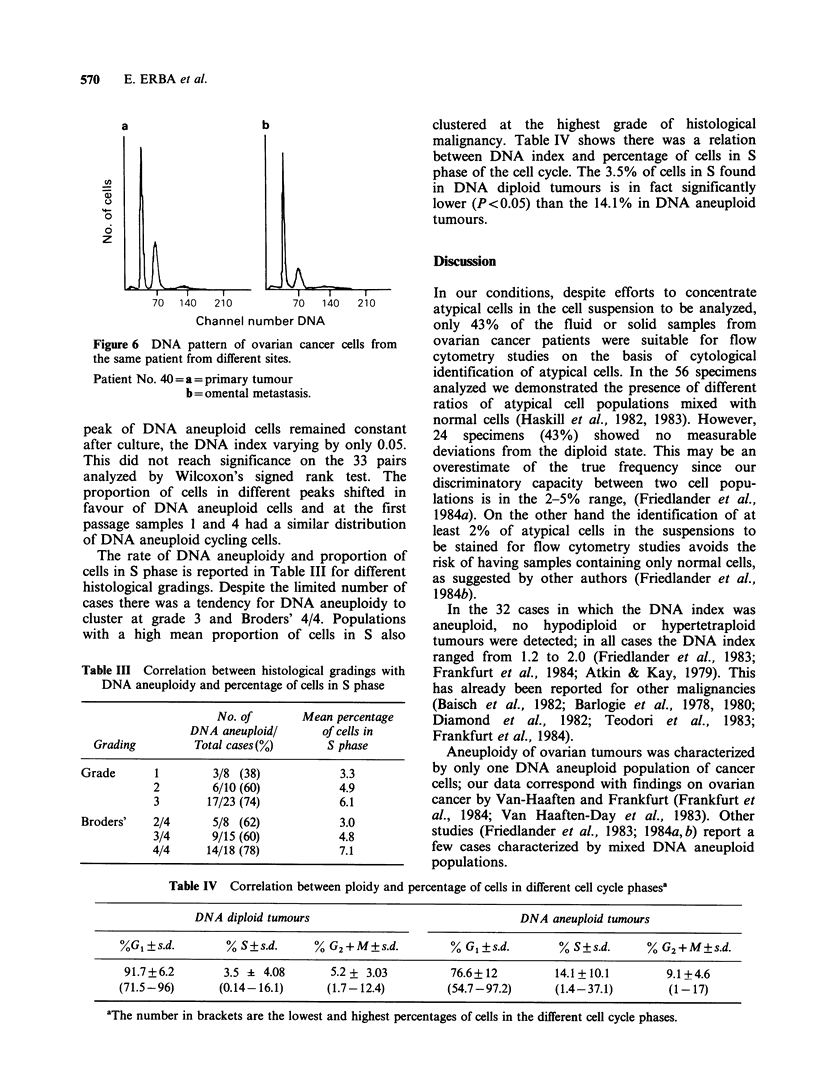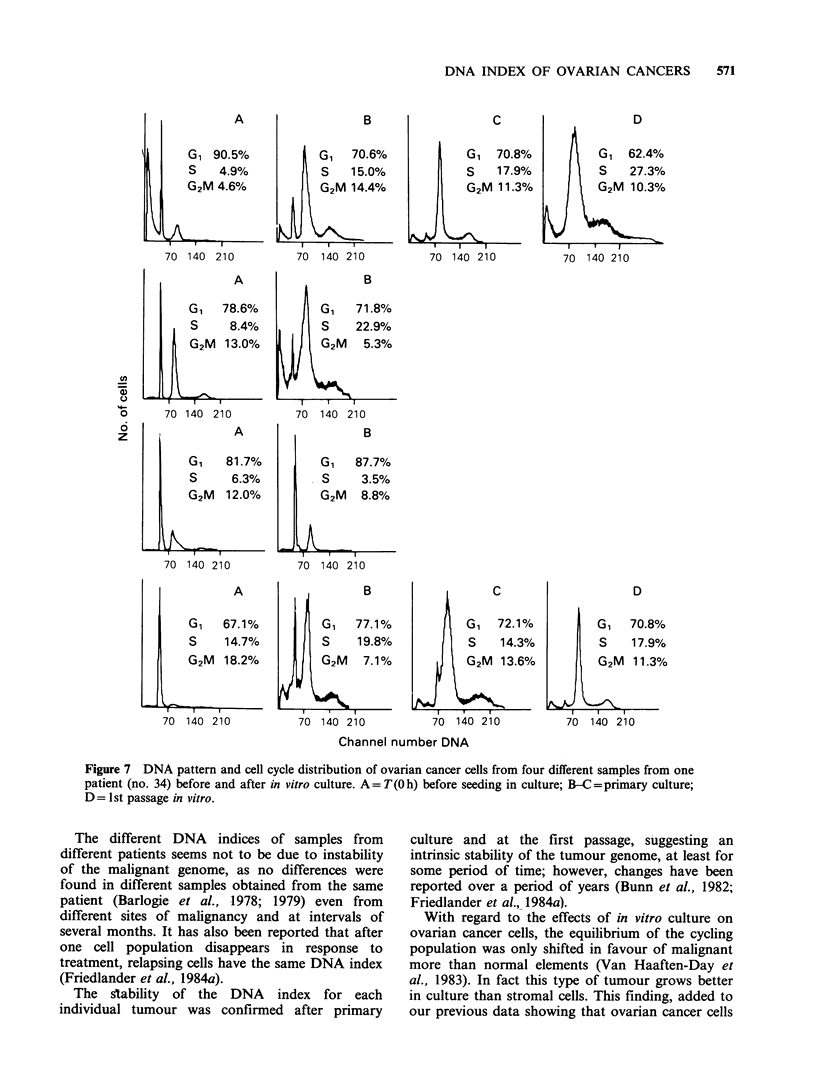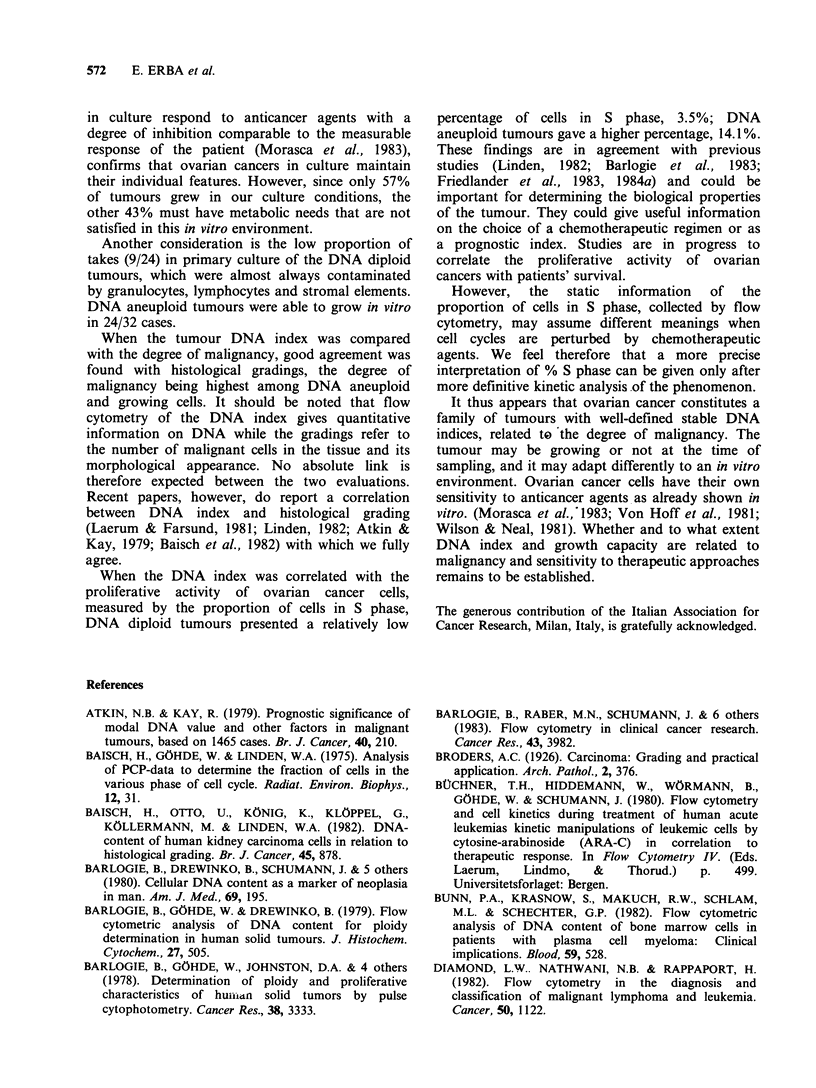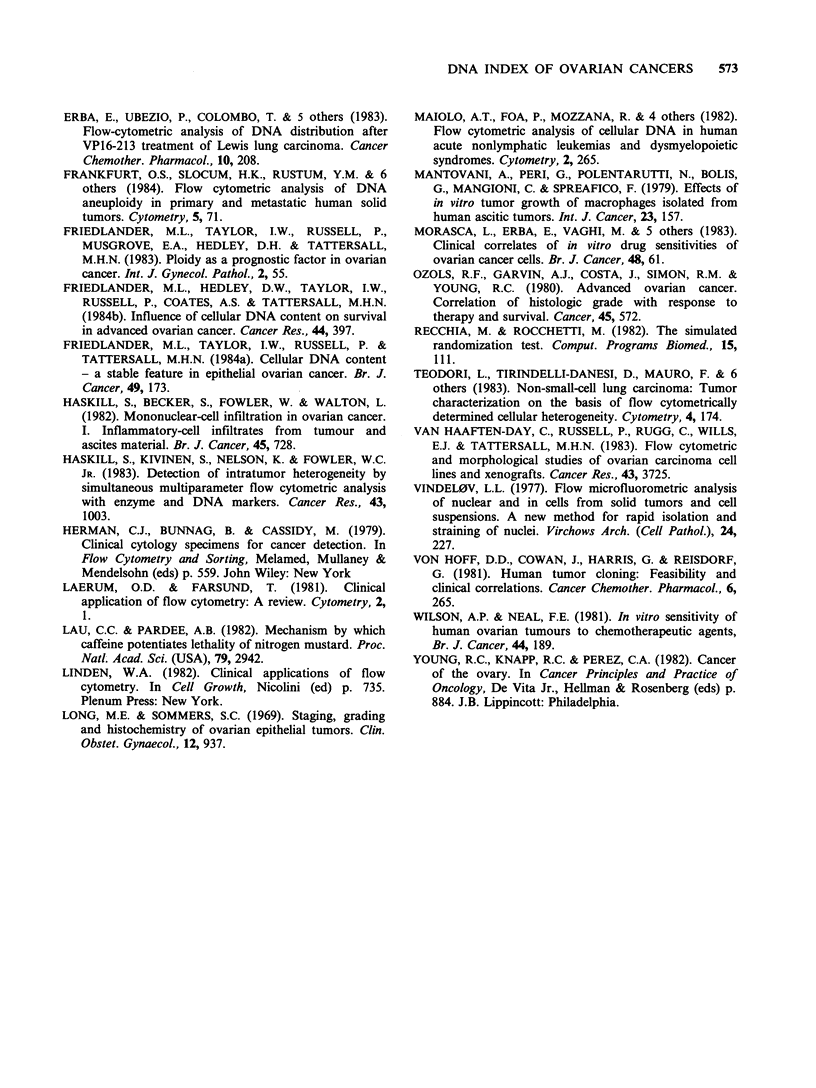# DNA index of ovarian carcinomas from 56 patients: in vivo in vitro studies.

**DOI:** 10.1038/bjc.1985.228

**Published:** 1985-10

**Authors:** E. Erba, M. Vaghi, S. Pepe, G. Amato, M. Bistolfi, P. Ubezio, C. Mangioni, F. Landoni, L. Morasca

## Abstract

Out of 130 ovarian cancer patients the DNA index of cells from ovarian carcinoma was studied in 56 cases in which cytospin preparations showed the presence of atypical cells. In 24 patients the population had a diploid DNA index (1.0) and in the others the DNA index ranged from 1.2 to 2.0 (tetraploid). No hypodiploid or hypertetraploid populations were detected. Repeated samples from the same patients did not show any significant differences and primary culture did not alter the DNA index. In contrast, cell cycle phase distribution differed greatly from sample to sample, as also the ratio between DNA diploid and DNA aneuploid populations. Primary culture was successful in 57% of the tumours, with a higher percentage of success in DNA aneuploid tumours. After primary culture the ratio between DNA aneuploid cells and DNA diploid cells increased. In relation to the histological gradings of malignancy, DNA aneuploid cells clustered in the highest grade of malignancy. The mean S-phase for tumours with a DNA index of 1.0 was 3.5 and 14.1% for those with DNA index greater than 1. Ovarian carcinomas show a large difference in DNA index between patients even after primary culture.


Br. J. Cancer (1985), 52, 565–573

### DNA index of ovarian carcinomas from 56 patients: *in vivo in vitro* studies

E. Erba[1], M. Vaghi[1], S. Pepe[1], G. Amato[1], M. Bistolfi[1], P. Ubezio[1], C. Mangioni[2], F. Landoni[2] & L. Morasca[1]

[1]*Istituto di Ricerche Farmacologiche 'Mario Negri' Via Eritrea, 62, 20157 Milan; and* [2]*Department of Obstetrics and Gynecology, S. Gerardo Hospital, Monza, Italy*

**Summary** Out of 130 ovarian cancer patients the DNA index of cells from ovarian carcinoma was studied in 56 cases in which cytospin preparations showed the presence of atypical cells. In 24 patients the population had a diploid DNA index (1.0) and in the others the DNA index ranged from 1.2 to 2.0 (tetraploid). No hypodiploid or hypertetraploid populations were detected. Repeated samples from the same patients did not show any significant differences and primary culture did not alter the DNA index. In contrast, cell cycle phase distribution differed greatly from sample to sample, as also the ratio between DNA diploid and DNA aneuploid populations. Primary culture was successful in 57% of the tumours, with a higher percentage of success in DNA aneuploid tumours. After primary culture the ratio between DNA aneuploid cells and DNA diploid cells increased. In relation to the histological gradings of malignancy, DNA aneuploid cells clustered in the highest grade of malignancy. The mean S-phase for tumours with a DNA index of 1.0 was 3.5 and 14.1% for those with DNA index >1. Ovarian carcinomas show a large difference in DNA index between patients even after primary culture.

Flow cytometric measurement of DNA content in human cancer cells is becoming a procedure for defining the DNA index and cell cycle distribution of the cell population. This may have several practical applications including prognostic value (Herman *et al.*, 1979; Laerum & Farsund, 1981; Linden, 1982) assessment of sensitivity to chemotherapy (Laerum & Farsund, 1981; Linden, 1982; Barlogie *et al.*, 1983; Buchner *et al.*, 1980) and the follow up of cultured tumours (Maiolo *et al.*, 1982; Friedlander *et al.*, 1983).

We applied the cytofluorometric technique to the study of human ovarian tumours with the following aims: (i) to describe the DNA index in ovarian cancers, (ii) to establish the extent to which the DNA index is modified by growing cancer cells *in vitro,* (iii) to correlate the DNA index with the cytological degree of malignancy.

### Materials and methods

Biopsy material for analysis was obtained from primary surgery, from second look laparotomy, pleural effusion or from ascitic fluid after paracentesis. All patients were classified by FIGO staging as III and IV and by histologically confirmed diagnosis as serous, mucinous, undifferentiated, mixed or endometroid ovarian carcinoma (Young *et al.*, 1982). Histological grading of tumours was evaluated in terms of well differentiated (grade 1), moderately differentiated (grade 2) and poorly differentiated (grade 3) (Long & Sommers, 1969) and according to Broders criteria (Broders, 1926; Ozols *et al.*, 1980). As cell suspensions were also used for seeding primary cultures, single cells were obtained from the biopsy material by a sterile procedure.

Tumour biopsy specimens were collected in PBS containing 100 U ml$^{-1}$ penicillin and 100 $\mu$g ml$^{-1}$ streptomycin (GIBCO Europe, Glasgow, Scotland) and rapidly processed in the laboratory a few hours after collection. Tissue was divided up and fragments were squashed on prestained slides (Test Simplets®, Boehringer, Mannheim GmbH, W. Germany) to test for the presence of cells spilled out from connective tissue and to confirm diagnosis grossly. Soft tissue from which cells spilled out easily was then disaggregated by treating 2 mm fragments with 0.25% tripsin 1:250 (Difco Laboratories, Detroit, Michigan, USA) in PBS without calcium and magnesium for 30 min at 37°C in a baffled flask on a magnetic stirrer.

Hard tumours from which few cells could be spilled out were minced carefully into very small fragments then treated for 1 h at 37°C with collagenase type 1 (Sigma Chemical Company, St. Louis, USA) dissolved 0.1–0.3% in medium 199 without serum. Cell suspensions were then washed and resuspended in growth medium.

Correspondence: E. Erba.
Received 20 November 1984; and in revised form, 14 May 1985.

© The Macmillan Press Ltd., 1985

Ascitic fluid was collected in heparinized bottles and centrifuged at 200 g for 10 min to separate the cellular phase. After resuspension in PBS a first microscopy check was made. Suspensions containing RBC and mononuclear cells were separated by a discontinuous gradient of 100% Ficoll-Hypaque (d = 1.077; MSL, Eurobio, Paris) for 20 min at 600 g. The tumour cells in the upper layer of the gradient were then freed of macrophages by the adhesion method (Mantovani *et al.*, 1979).

When a large number of lymphocytes was present, a second gradient of one 75% and one 100% Fycoll-Hypaque was made to separate cancer cells from lymphocytes. After this step ascitic cells were resuspended in growth medium.

At this stage viability was tested by the erythrosine dye exclusion test in cell suspensions from solid or ascitic samples. A cytospin preparation of each sample was also made to check the presence of different types of normal and neoplastic cells before staining for flow cytometry. Only suspensions positive for atypical elements and with >70% viable cells were then seeded at 350,000 viable cells $ml^{-1}$ in T25 $cm^2$ tissue culture flasks (Sterilin-Flow Laboratories, Irvine, UK). Growth medium 199 was supplemented with 15% foetal calf serum, 2 mM glutamine, 6% of MEM essential aminoacid stock solution, 3% stock solution of MEM vitamins (all purchased from Flow Laboratories, Irvine, UK) and 20 mM HEPES; (Merck, Darmstadt, W. Germany). The pH was set at 7.2 in air, with osmotic pressure kept at $285 \pm 10$ mosmol (Morasca *et al.*, 1983). Successful cultures gave monolayers in T-flasks and were used for *in vitro* testing of sensitivity to anticancer agents, when there were sufficient cells present in suspension to run enough primary cultures.

The samples for flow cytometry were stained with propidium iodide (PI) (Calbiochem Behring Co., USA) by adding 3 ml of PI solution (50 $\mu g\,ml^{-1}$ PI in 0.1% sodium citrate containing 30 $\mu l$ Nonidet P 40 detergent (Sigma), and 30 $\mu l$ RNAse 0.5 mg $ml^{-1}$ (Calbiochem, Behring, Co., USA), to 200–300 $\mu l$ of cell suspension at room temperature for 30–45 min (Vindeløv, 1977). Cells in culture were washed three times with PBS and directly stained with the same PI solution. In this way nuclei were dislodged from the cells, adhering to the plastic surface of the flask, and entered into suspension without the cells having to be suspended (Lau & Pardee, 1982). The suitability of the preparation, the specificity of staining and the absence of aggregates were checked by fluorescence microscopy before the sample was run.

To determine the DNA index, human leucocytes from freshly collected blood were used as standard. Standard was run before and after the sample to check for drifting of the laser output. Doublets were <1% by morphological examination of the tumour cell suspension. Leucocyte standards always contained <0.8% of doublets.

Cytofluorometric analysis was performed using a 30L Cytofluorograph (Ortho Instruments, USA). The fluorescence pulses were detected in a spectral range between 580 and 750 nm (to exclude the overlapping region of excitation and emission spectra or unbound PI), and integrated. The coefficient of variation (CV) of the standard was between 1.5–2.5% while in ovarian cancer cells the CV of the $G_0/G_1$ peak was 3–4%. Routinely at least 50,000–100,000 cells were measured at a flow rate of less than 500 cells $sec^{-1}$ (Erba *et al.*, 1983). Ploidy was expressed as DNA index, representing the ratio between the $G_1$ peak of ovarian cancer cells and the peak of leucocytes (Barlogie *et al.*, 1983). The percentage of cells in the cell cycle phases was calculated by the method of Baisch *et al.* (1975) and the number of cells in each phase was also calculated. The simulated randomization test was used to study the relationship between ploidy and the percentage of cells in the S phase (Recchia & Rocchetti, 1982).

### Results

Only 56 samples out of 130 could be studied by flow cytometry on the basis of morphological evidence of atypical cells in the suspensions. Among these we found ten patients (1–10) presenting a DNA distribution identical to normal human leucocytes, with a DNA index of 1.0 and no cycling cells. Despite the absence of cycling cells, in four of these cases cells grew *in vitro*. In all these samples atypical cells were present in cytospin preparations, and only in two of them was the percentage of atypical cells <5%. In two cases with 26 and 30% of atypical cells, however, we were not successful in growing cells *in vitro*.

DNA diploid cycling cells were found in 14 patients (11–24) (Table I); the proportion of cells in S phase ranged from the threshold of detection (0.2%) to 16.1%. In this group of DNA diploid cells, the capacity to grow *in vitro* was low (35%). Figure 1 shows a typical histogram (patient No. 14).

Non cycling cells were found in a few DNA aneuploid tumours. Patients 25, 26 and 27 with DNA indices of 1.2, 1.89 and 2.00 respectively, had DNA distributions with no detectable cells in the S and $G_2 + M$ compartments. Two of these tumours nevertheless grew *in vitro*. Figure 2 represents the DNA histogram of patient No. 25.

Table II reports eighteen cases (28–45) in which two different cell populations were detected: a

**Table I** Fourteen cases of ovarian cancer with cycling DNA diploid tumour cells[a]

| [a]*Patient No.* | *DNA index* | *Cell cycle phase %* $G_1$ | *S* | $G_2M$ | *Growth in culture* | *Description*[b] | |
|---|---|---|---|---|---|---|---|
| 11 | 1 | 94.4 | 0.6 | 5 | – | UN | A |
| 12 | 1 | 95.5 | 2.4 | 2 | + | SE | A |
| 13 | 1 | 94.1 | 4.2 | 1.7 | – | SE | A |
| 14 | 1 | 88 | 1.6 | 10.4 | – | MU | P |
| 15 | 1 | 92.5 | 3.1 | 4.4 | Fibroblasts | MX | P |
| 16 | 1 | 91.2 | 0.6 | 8.2 | – | EN | P |
| 17 | 1 | 90.4 | 4.3 | 5.3 | – | UN | A |
| 18 | 1 | 93.2 | 1.6 | 5.1 | + | SE | A |
| 19 | 1 | 96 | 0.2 | 3.8 | – | EN | P |
| 20 | 1 | 95.8 | 1.3 | 2.9 | + | SE | A |
| 21 | 1 | 87.7 | 7.1 | 5.2 | – | SE | P |
| 22 | 1 | 71.5 | 16.1 | 12.4 | – | SE | OM |
| 23 | 1 | 90.9 | 4.7 | 4.4 | + | SE | A |
| 24 | 1 | 93.9 | 2.3 | 3.8 | + | SE | P |

[a]DNA diploid Index = 1.0.

The percentages of cells in $G_1$, S and $G_2+M$ phases of the cell cycle, success in culture, the histological type and origin of cancer cells are indicated; [b]UN = Undifferentiated, SE = Serous, MU = Mucinous, MX = Mixed, EN = Endometrioid, A = Ascitic fluid, P = Primary tumour, OM = Omental metastasis.

**Figure 1** DNA diploid index and cell cycle distribution of patient No. 14. From left to right, the first peak represents the $G_0/G_1$ peak of DNA diploid cancer cells overlapping with human leucocytes; the small second peak represents the tumour cells in $G_2+M$ phase of the cell cycle; between $G_0/G_1$ and $G_2+M$ peaks are cells in S phase.

**Figure 2** DNA aneuploid index of patient No. 25. From left to right, the first peak represents the DNA diploid cell in the tumour overlapping with human leucocytes used as standard; the second peak represents the DNA aneuploid cancer cells.

DNA diploid non-cycling and a DNA aneuploid cycling population. In these cases the cells in S phase ranged from 1.1 to 37.1% and 15 out of 18 of them gave primary cultures. Figure 3 shows a DNA histogram of one of these cases (patient No. 36).

The last eleven patients (45–56) had tumours with a complex DNA distribution suggesting the

**Table II** Eighteen cases of ovarian cancer with cycling DNA aneuploid tumour cells[a]

| [a]*Patient No.* | *DNA index* | *Cell cycle phase %* $G_1$ | *S* | $G_2M$ | *Growth in culture* | *Description*[b] | |
|---|---|---|---|---|---|---|---|
| 28 | 1.48 | 97.2 | 1.4 | 1.4 | – | EN | P |
| 29 | 1.4 | 85.3 | 4.7 | 1 | + | SE | PE |
| 30 | 1.38 | 69.9 | 17.2 | 12.9 | Fibroblasts | SE | PE |
| 31 | 1.52 | 59.8 | 23.2 | 16.9 | + | SE | P |
| 32 | 1.60 | 68.5 | 24 | 7.5 | + | SE | A |
| 33 | 1.72 | 85.5 | 8.9 | 5.6 | + | SE | P |
| 34 | 1.74 | 83.2 | 9.2 | 7.6 | + | SE | A |
| 35 | 1.67 | 79.8 | 7.3 | 12.9 | + | SE | A |
| 36 | 1.72 | 78.6 | 8.4 | 13 | + | SE | A |
| 37 | 1.94 | 93.2 | 6.8 | 0 | + | SE | P |
| 38 | 1.27 | 93.3 | 1.1 | 5.6 | – | SE | A |
| 39 | 1.50 | 67.1 | 21.2 | 11.7 | + | SE | A |
| 40 | 1.87 | 60.2 | 30.8 | 9 | + | SE | A |
| 41 | 1.19 | 68 | 15 | 17 | + | SE | A |
| 42 | 1.72 | 54.7 | 37.1 | 8.2 | + | SE | A |
| 43 | 1.30 | 74.8 | 11.9 | 13.3 | + | SE | A |
| 44 | 1.35 | 69.9 | 17.1 | 15.8 | + | SE | A |
| 45 | 1.2 | 90 | 5.5 | 4.5 | – | EN | P |

[a]The DNA index, cell cycle distribution, growth in culture, tumour histological type and origin of cancer cells are reported; [b]EN = Endometrioid, SE = Serous, P = Primary tumour, PE = Pleural effusion, A = Ascitic fluid.

**Figure 3** DNA aneuploid index and cell cycle distribution of patient No. 36. The left-hand peak represents the $G_0/G_1$ peak of DNA diploid cells overlapping with human leukocytes; the right-hand peak, the $G_1$ peak of DNA aneuploid cancer cells with cells in S and $G_2+M$ phases of the cell cycle.

presence of two cycling populations, as can be seen from the DNA distribution of patient No. 48 (Figure 4). In these cases no attempt was made to extrapolate a cell cycle distribution because the portion of DNA diploid cells in $G_2+M$ phase overlapped the S phase of DNA aneuploid cells. DNA indices ranged from 1.39 to 1.89 and 7/11 samples grew in culture.

**Figure 4** DNA pattern of patient No. 48: two different cycling cell populations are present, one DNA diploid, the higher peak on the left, and one DNA aneuploid, the second peak.

From a few patients it was possible to collect several samples to check the stability of the DNA index over time and from different lesions. Figure 5 illustrates the histograms of two patients: patient No. 33 over a 4-month period had the DNA aneuploid population stable on channel 85 while the ratio of DNA diploid to DNA aneuploid cells and cell cycle phase distribution differed markedly from one sample to the next.

Patient No. 44 was monitored over 6 months with ten samples of ascitic fluid. The DNA index remained stable in this case also. Figure 6 shows one of the cases in which primary and metastatic tumours revealed no difference in DNA index.

The DNA index and the cell cycle distribution was studied before and after *in vitro* culture in 33 of the 56 cases studied (57%). Figure 7 reports four samples of ascitic fluid from a single patient. The

**Figure 5** DNA pattern and cell cycle distribution of samples from two patients, from whom several samples could be collected at different times.
*Patient no. 33*: samples A–B, D–H = ascitic fluid; C = Pleural effusion.
*Patient no. 44*: all samples = Ascitic fluid.

**Figure 6** DNA pattern of ovarian cancer cells from the same patient from different sites.

Patient No. 40 = **a** = primary tumour
**b** = omental metastasis.

peak of DNA aneuploid cells remained constant after culture, the DNA index varying by only 0.05. This did not reach significance on the 33 pairs analyzed by Wilcoxon's signed rank test. The proportion of cells in different peaks shifted in favour of DNA aneuploid cells and at the first passage samples 1 and 4 had a similar distribution of DNA aneuploid cycling cells.

The rate of DNA aneuploidy and proportion of cells in S phase is reported in Table III for different histological gradings. Despite the limited number of cases there was a tendency for DNA aneuploidy to cluster at grade 3 and Broders' 4/4. Populations with a high mean proportion of cells in S also clustered at the highest grade of histological malignancy. Table IV shows there was a relation between DNA index and percentage of cells in S phase of the cell cycle. The 3.5% of cells in S found in DNA diploid tumours is in fact significantly lower ($P<0.05$) than the 14.1% in DNA aneuploid tumours.

**Table III** Correlation between histological gradings with DNA aneuploidy and percentage of cells in S phase

| *Grading* | | *No. of DNA aneuploid/ Total cases (%)* | *Mean percentage of cells in S phase* |
|---|---|---|---|
| Grade | 1 | 3/8 (38) | 3.3 |
| | 2 | 6/10 (60) | 4.9 |
| | 3 | 17/23 (74) | 6.1 |
| Broders' | 2/4 | 5/8 (62) | 3.0 |
| | 3/4 | 9/15 (60) | 4.8 |
| | 4/4 | 14/18 (78) | 7.1 |

### Discussion

In our conditions, despite efforts to concentrate atypical cells in the cell suspension to be analyzed, only 43% of the fluid or solid samples from ovarian cancer patients were suitable for flow cytometry studies on the basis of cytological identification of atypical cells. In the 56 specimens analyzed we demonstrated the presence of different ratios of atypical cell populations mixed with normal cells (Haskill *et al.*, 1982, 1983). However, 24 specimens (43%) showed no measurable deviations from the diploid state. This may be an overestimate of the true frequency since our discriminatory capacity between two cell populations is in the 2–5% range, (Friedlander *et al.*, 1984*a*). On the other hand the identification of at least 2% of atypical cells in the suspensions to be stained for flow cytometry studies avoids the risk of having samples containing only normal cells, as suggested by other authors (Friedlander *et al.*, 1984*b*).

In the 32 cases in which the DNA index was aneuploid, no hypodiploid or hypertetraploid tumours were detected; in all cases the DNA index ranged from 1.2 to 2.0 (Friedlander *et al.*, 1983; Frankfurt *et al.*, 1984; Atkin & Kay, 1979). This has already been reported for other malignancies (Baisch *et al.*, 1982; Barlogie *et al.*, 1978, 1980; Diamond *et al.*, 1982; Teodori *et al.*, 1983; Frankfurt *et al.*, 1984).

Aneuploidy of ovarian tumours was characterized by only one DNA aneuploid population of cancer cells; our data correspond with findings on ovarian cancer by Van-Haaften and Frankfurt (Frankfurt *et al.*, 1984; Van Haaften-Day *et al.*, 1983). Other studies (Friedlander *et al.*, 1983; 1984*a,b*) report a few cases characterized by mixed DNA aneuploid populations.

**Table IV** Correlation between ploidy and percentage of cells in different cell cycle phases[a]

| *DNA diploid tumours* | | | *DNA aneuploid tumours* | | |
|---|---|---|---|---|---|
| *%$G_1$ ± s.d.* | *% S ± s.d.* | *% $G_2+M$ ± s.d.* | *% $G_1$ ± s.d.* | *% S ± s.d.* | *% $G_2+M$ ± s.d.* |
| 91.7 ± 6.2 | 3.5 ± 4.08 | 5.2 ± 3.03 | 76.6 ± 12 | 14.1 ± 10.1 | 9.1 ± 4.6 |
| (71.5 – 96) | (0.14 – 16.1) | (1.7 – 12.4) | (54.7 – 97.2) | (1.4 – 37.1) | (1 – 17) |

[a]The number in brackets are the lowest and highest percentages of cells in the different cell cycle phases.

**Figure 7** DNA pattern and cell cycle distribution of ovarian cancer cells from four different samples from one patient (no. 34) before and after *in vitro* culture. A = $T(0\,h)$ before seeding in culture; B–C = primary culture; D = 1st passage *in vitro*.

The different DNA indices of samples from different patients seems not to be due to instability of the malignant genome, as no differences were found in different samples obtained from the same patient (Barlogie *et al.*, 1978; 1979) even from different sites of malignancy and at intervals of several months. It has also been reported that after one cell population disappears in response to treatment, relapsing cells have the same DNA index (Friedlander *et al.*, 1984*a*).

The stability of the DNA index for each individual tumour was confirmed after primary culture and at the first passage, suggesting an intrinsic stability of the tumour genome, at least for some period of time; however, changes have been reported over a period of years (Bunn *et al.*, 1982; Friedlander *et al.*, 1984*a*).

With regard to the effects of *in vitro* culture on ovarian cancer cells, the equilibrium of the cycling population was only shifted in favour of malignant more than normal elements (Van Haaften-Day *et al.*, 1983). In fact this type of tumour grows better in culture than stromal cells. This finding, added to our previous data showing that ovarian cancer cells in culture respond to anticancer agents with a degree of inhibition comparable to the measurable response of the patient (Morasca *et al.*, 1983), confirms that ovarian cancers in culture maintain their individual features. However, since only 57% of tumours grew in our culture conditions, the other 43% must have metabolic needs that are not satisfied in this *in vitro* environment.

Another consideration is the low proportion of takes (9/24) in primary culture of the DNA diploid tumours, which were almost always contaminated by granulocytes, lymphocytes and stromal elements. DNA aneuploid tumours were able to grow *in vitro* in 24/32 cases.

When the tumour DNA index was compared with the degree of malignancy, good agreement was found with histological gradings, the degree of malignancy being highest among DNA aneuploid and growing cells. It should be noted that flow cytometry of the DNA index gives quantitative information on DNA while the gradings refer to the number of malignant cells in the tissue and its morphological appearance. No absolute link is therefore expected between the two evaluations. Recent papers, however, do report a correlation between DNA index and histological grading (Laerum & Farsund, 1981; Linden, 1982; Atkin & Kay, 1979; Baisch *et al.*, 1982) with which we fully agree.

When the DNA index was correlated with the proliferative activity of ovarian cancer cells, measured by the proportion of cells in S phase, DNA diploid tumours presented a relatively low percentage of cells in S phase, 3.5%; DNA aneuploid tumours gave a higher percentage, 14.1%. These findings are in agreement with previous studies (Linden, 1982; Barlogie *et al.*, 1983; Friedlander *et al.*, 1983, 1984*a*) and could be important for determining the biological properties of the tumour. They could give useful information on the choice of a chemotherapeutic regimen or as a prognostic index. Studies are in progress to correlate the proliferative activity of ovarian cancers with patients' survival.

However, the static information of the proportion of cells in S phase, collected by flow cytometry, may assume different meanings when cell cycles are perturbed by chemotherapeutic agents. We feel therefore that a more precise interpretation of % S phase can be given only after more definitive kinetic analysis of the phenomenon.

It thus appears that ovarian cancer constitutes a family of tumours with well-defined stable DNA indices, related to the degree of malignancy. The tumour may be growing or not at the time of sampling, and it may adapt differently to an *in vitro* environment. Ovarian cancer cells have their own sensitivity to anticancer agents as already shown *in vitro*. (Morasca *et al.*, 1983; Von Hoff *et al.*, 1981; Wilson & Neal, 1981). Whether and to what extent DNA index and growth capacity are related to malignancy and sensitivity to therapeutic approaches remains to be established.

The generous contribution of the Italian Association for Cancer Research, Milan, Italy, is gratefully acknowledged.

### References

ATKIN, N.B. & KAY, R. (1979). Prognostic significance of modal DNA value and other factors in malignant tumours, based on 1465 cases. *Br. J. Cancer*, **40**, 210.

BAISCH, H., GÖHDE, W. & LINDEN, W.A. (1975). Analysis of PCP-data to determine the fraction of cells in the various phase of cell cycle. *Radiat. Environ. Biophys.*, **12**, 31.

BAISCH, H., OTTO, U., KÖNIG, K., KLÖPPEL, G., KÖLLERMANN, M. & LINDEN, W.A. (1982). DNA-content of human kidney carcinoma cells in relation to histological grading. *Br. J. Cancer*, **45**, 878.

BARLOGIE, B., DREWINKO, B., SCHUMANN, J. & 5 others (1980). Cellular DNA content as a marker of neoplasia in man. *Am. J. Med.*, **69**, 195.

BARLOGIE, B., GÖHDE, W. & DREWINKO, B. (1979). Flow cytometric analysis of DNA content for ploidy determination in human solid tumours. *J. Histochem. Cytochem.*, **27**, 505.

BARLOGIE, B., GÖHDE, W., JOHNSTON, D.A. & 4 others (1978). Determination of ploidy and proliferative characteristics of human solid tumors by pulse cytophotometry. *Cancer Res.*, **38**, 3333.

BARLOGIE, B., RABER, M.N., SCHUMANN, J. & 6 others (1983). Flow cytometry in clinical cancer research. *Cancer Res.*, **43**, 3982.

BRODERS, A.C. (1926). Carcinoma: Grading and practical application. *Arch. Pathol.*, **2**, 376.

BÜCHNER, T.H., HIDDEMANN, W., WÖRMANN, B., GÖHDE, W. & SCHUMANN, J. (1980). Flow cytometry and cell kinetics during treatment of human acute leukemias kinetic manipulations of leukemic cells by cytosine-arabinoside (ARA-C) in correlation to therapeutic response. In *Flow Cytometry IV*. (Eds. Laerum, Lindmo, & Thorud.) p. 499. Universitetsforlaget: Bergen.

BUNN, P.A., KRASNOW, S., MAKUCH, R.W., SCHLAM, M.L. & SCHECHTER, G.P. (1982). Flow cytometric analysis of DNA content of bone marrow cells in patients with plasma cell myeloma: Clinical implications. *Blood*, **59**, 528.

DIAMOND, L.W.. NATHWANI, N.B. & RAPPAPORT, H. (1982). Flow cytometry in the diagnosis and classification of malignant lymphoma and leukemia. *Cancer*, **50**, 1122.

ERBA, E., UBEZIO, P., COLOMBO, T. & 5 others (1983). Flow-cytometric analysis of DNA distribution after VP16-213 treatment of Lewis lung carcinoma. *Cancer Chemother. Pharmacol.*, **10**, 208.

FRANKFURT, O.S., SLOCUM, H.K., RUSTUM, Y.M. & 6 others (1984). Flow cytometric analysis of DNA aneuploidy in primary and metastatic human solid tumors. *Cytometry*, **5**, 71.

FRIEDLANDER, M.L., TAYLOR, I.W., RUSSELL, P., MUSGROVE, E.A., HEDLEY, D.H. & TATTERSALL, M.H.N. (1983). Ploidy as a prognostic factor in ovarian cancer. *Int. J. Gynecol. Pathol.*, **2**, 55.

FRIEDLANDER, M.L., HEDLEY, D.W., TAYLOR, I.W., RUSSELL, P., COATES, A.S. & TATTERSALL, M.H.N. (1984b). Influence of cellular DNA content on survival in advanced ovarian cancer. *Cancer Res.*, **44**, 397.

FRIEDLANDER, M.L., TAYLOR, I.W., RUSSELL, P. & TATTERSALL, M.H.N. (1984a). Cellular DNA content – a stable feature in epithelial ovarian cancer. *Br. J. Cancer*, **49**, 173.

HASKILL, S., BECKER, S., FOWLER, W. & WALTON, L. (1982). Mononuclear-cell infiltration in ovarian cancer. I. Inflammatory-cell infiltrates from tumour and ascites material. *Br. J. Cancer*, **45**, 728.

HASKILL, S., KIVINEN, S., NELSON, K. & FOWLER, W.C. JR. (1983). Detection of intratumor heterogeneity by simultaneous multiparameter flow cytometric analysis with enzyme and DNA markers. *Cancer Res.*, **43**, 1003.

HERMAN, C.J., BUNNAG, B. & CASSIDY, M. (1979). Clinical cytology specimens for cancer detection. In *Flow Cytometry and Sorting*, Melamed, Mullaney & Mendelsohn (eds) p. 559. John Wiley: New York

LAERUM, O.D. & FARSUND, T. (1981). Clinical application of flow cytometry: A review. *Cytometry*, **2**, 1.

LAU, C.C. & PARDEE, A.B. (1982). Mechanism by which caffeine potentiates lethality of nitrogen mustard. *Proc. Natl. Acad. Sci.* (USA), **79**, 2942.

LINDEN, W.A. (1982). Clinical applications of flow cytometry. In *Cell Growth*, Nicolini (ed) p. 735. Plenum Press: New York.

LONG, M.E. & SOMMERS, S.C. (1969). Staging, grading and histochemistry of ovarian epithelial tumors. *Clin. Obstet. Gynaecol.*, **12**, 937.

MAIOLO, A.T., FOA, P., MOZZANA, R. & 4 others (1982). Flow cytometric analysis of cellular DNA in human acute nonlymphatic leukemias and dysmyelopoietic syndromes. *Cytometry*, **2**, 265.

MANTOVANI, A., PERI, G., POLENTARUTTI, N., BOLIS, G., MANGIONI, C. & SPREAFICO, F. (1979). Effects of *in vitro* tumor growth of macrophages isolated from human ascitic tumors. *Int. J. Cancer*, **23**, 157.

MORASCA, L., ERBA, E., VAGHI, M. & 5 others (1983). Clinical correlates of *in vitro* drug sensitivities of ovarian cancer cells. *Br. J. Cancer*, **48**, 61.

OZOLS, R.F., GARVIN, A.J., COSTA, J., SIMON, R.M. & YOUNG, R.C. (1980). Advanced ovarian cancer. Correlation of histologic grade with response to therapy and survival. *Cancer*, **45**, 572.

RECCHIA, M. & ROCCHETTI, M. (1982). The simulated randomization test. *Comput. Programs Biomed.*, **15**, 111.

TEODORI, L., TIRINDELLI-DANESI, D., MAURO, F. & 6 others (1983). Non-small-cell lung carcinoma: Tumor characterization on the basis of flow cytometrically determined cellular heterogeneity. *Cytometry*, **4**, 174.

VAN HAAFTEN-DAY, C., RUSSELL, P., RUGG, C., WILLS, E.J. & TATTERSALL, M.H.N. (1983). Flow cytometric and morphological studies of ovarian carcinoma cell lines and xenografts. *Cancer Res.*, **43**, 3725.

VINDELØV, L.L. (1977). Flow microfluorometric analysis of nuclear and in cells from solid tumors and cell suspensions. A new method for rapid isolation and straining of nuclei. *Virchows Arch.* (*Cell Pathol.*), **24**, 227.

VON HOFF, D.D., COWAN, J., HARRIS, G. & REISDORF, G. (1981). Human tumor cloning: Feasibility and clinical correlations. *Cancer Chemother. Pharmacol.*, **6**, 265.

WILSON, A.P. & NEAL, F.E. (1981). *In vitro* sensitivity of human ovarian tumours to chemotherapeutic agents, *Br. J. Cancer*, **44**, 189.

YOUNG, R.C., KNAPP, R.C. & PEREZ, C.A. (1982). Cancer of the ovary. In *Cancer Principles and Practice of Oncology*, De Vita Jr., Hellman & Rosenberg (eds) p. 884. J.B. Lippincott: Philadelphia.